# A case report of palmoplantar keratoderma in a 3-year-old girl: A structured approach in primary care settings

**DOI:** 10.51866/cr.668

**Published:** 2025-07-09

**Authors:** Asma Amirah Mohd Noor, Azwanis Abdul Hadi, Abdul Rahman Che Abdul Rahim

**Affiliations:** 1 MBChB, MMed (Family Medicine), Department of Family Medicine, Kulliyyah of Medicine, International Islamic University Malaysia, Kuantan, Pahang, Malaysia. E-mail: azwanis@iium.edu.my; 2 MBBS, Department of Family Medicine, Kulliyyah of Medicine, International Islamic University Malaysia, Kuantan, Pahang, Malaysia.; 3 MBBS, MRCP, Adv MDerm, Department of Dermatology, Hospital Tengku Ampuan Afzan, Kuantan, Pahang, Malaysia.

**Keywords:** Palmoplantar, Keratoderma, Genetic disease, Diagnosis, Primary health care

## Abstract

Palmoplantar keratoderma (PPK) is a dermatological disorder characterised by excessive thickening of the palms and soles, encompassing more than 20 conditions. The disease is often misdiagnosed in primary care settings, leading to unnecessary treatments and delays. We present the case of a 3-year-old girl with skin thickening on both her palms and soles persisting for 2 years, initially believed to be an acquired condition. Subsequent evaluation revealed a family history of similar skin lesions. This case report highlights the crucial role of family physicians in differentiating hereditary from acquired PPK, especially in settings where advanced testing is unavailable. Implementing a structured diagnostic approach at the primary care level can significantly improve patient management and reduce morbidities and healthcare costs. This case contributes to the existing knowledge in this field, where hereditary PPK remains underexplored.

## Introduction

Palmoplantar keratoderma (PPK) comprises a diverse spectrum of over 20 conditions,^1,2^ each with unique features and diagnostic challenges, particularly in resource-limited primary care clinics.^3^ These complexities are compounded by the under-recognition of PPK’s the actual incidence and prevalence.^1^ Acquired PPK is more common, estimated at 1 in 100 cases, while hereditary palmoplantar keratoderma (HPPK) is rare in Asia, with a prevalence of 1-3 per 10,000.^4^ Table 1 illustrate their distinguishing features.

**Table 1:** Distinguishing features between acquired and hereditary palmoplantar keratoderma (PPK).

Feature	Acquired PPK	Hereditary PPK
Aetiology	Secondary to underlying conditions^1,5^ such as: **Dermatologic conditions:** Psoriasis, hyperkeratotic eczema, lichen planus or pityriasis rubra pilaris **Systemic diseases:** Lupus erythematosus, Reiter’s syndrome, HIV or, thyroid abnormalities **Infections:** Norwegian scabies or, secondary syphilis **Malignancies:** Paraneoplastic syndrome in solid organ carcinomas or, Sezary syndrome **Drugs and chemicals:** Arsenic, halogens, verapamil, hydroxyurea or, bleomycin **Malnutrition**	Caused by genetic mutations; inheritance may be autosomal dominant, recessive, or sporadic^3^
Age of onset	Typically develops later in life^6^; may occur during infancy^5^	Often begins in childhood or infancy, although some forms may appear later^3^
Family history	Family history might be associated^5^	Family history is often positive,^3^ although sporadic cases due to de novo mutations may also occur
Distribution of lesions	The lesion distribution may differ^1^; additional clinical features such as psoriatic nail changes^5^	Symmetric distribution of lesions^1^
Attributed to friction	No^1^	Yes^3^
Treatment response	Improves with treatment of the underlying condition^1^	Often managed symptomatically with emollients and keratolytics; some forms may not improve significantly with treatment^1^
Prognosis	Good prognosis with appropriate management of the underlying causes^1^	Lifelong condition, although symptoms can be managed^1^

Most PPK cases are initially presumed to be acquired. Nevertheless, numerous hereditary classifications can be overwhelming. Herein, we report a case of diffuse HPPK from early infancy, which was initially misdiagnosed. We illustrate the effectiveness of good history-taking and examination at the primary care level in diagnosis without an advanced testing. Thus, the complexities of classifying HPPK can be eased.

## Case presentation

A 3-year-old Malay girl presented with skin thickening persisting for 2 years. Symptoms began when she started crawling and walking, with gradual thickening over both palms and soles. A general practitioner initially reassured her parents it was due to friction. However, her condition persisted despite various creams and oil-based treatments, which impacted her daily activities. The lesions remained dry and pruritic, resulting in cracked and sore skin. After water exposure, the affected areas demonstrated whitish and spongy. There was no itching or discharge. She had not experienced excessive sweating or exposure to chemicals. She was euthyroid, with good appetite and no significant medical history. She was not on medication. However, upon further history-taking, similar dermatological symptoms were discovered in her father and older brother.

**Figure 1:**
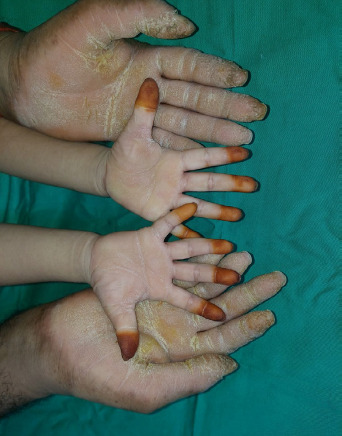
Diffuse epidermal thickening that involves the entire palms of both the patient and her father. A henna stain was also noted on all her distal fingers.

**Figure 2:**
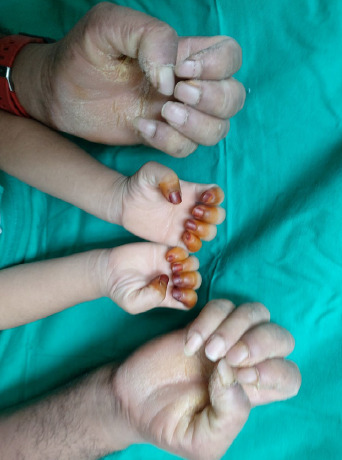
No nail dystrophy, knuckle pad or hyperhidrosis.

**Figure 3:**
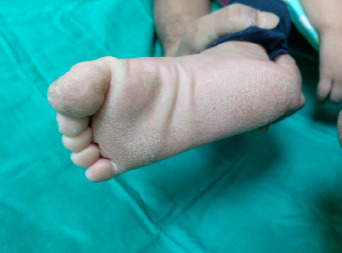
Diffuse epidermal thickening that involves the entire surface of the soles, including the arch.

**Figure 4:**
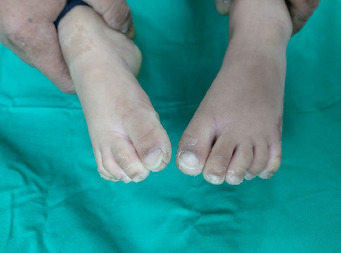
Transgrediens or hyperkeratosis extends onto the dorsal surface of the foot.

On examination, the child appeared neither dysmorphic nor cachexic. Her palms showed diffuse dryness, coarseness, and hyperkeratosis, with non-transgrediens (Figures 1-3) and henna staining noted on her fingernails. The soles exhibited similar features, but with transgrediens extension onto the dorsal feet (Figure 4). No signs of pseudo-ainhum, mutilation, waxy skin, knuckle pads, distant hyperkeratotic or scaly skin, nail dystrophies, woolly hair, skin hypo- or hyperpigmentation or hyperhidrosis were observed. There was neither lymphadenopathy nor apex beat displacement, and the gums appeared healthy.

A dermatology consultation confirmed hereditary diffuse PPK, based on the clinical presentation and family history. Differential diagnoses such as eczema, psoriasis, lichen planus, pityriasis rubra pilarise infective dermatosis, trauma, drugs, chemicals and malignancies were excluded. The parents were informed of the diagnosis and prognosis.

Treatment with urea 10% cream LA BD and Vaseline LA QID was started. Within months, skin thickening significantly improved. The parents were informed about the possibility of temporary improvement and hereditary risk.

## Discussion

HPPK constitutes a heterogeneous group of keratinisation disorders, primarily marked by excessive epidermal thickening.^3^ In our case, clinical phenotyping and family history were key to diagnosis. Categorising HPPK is challenging due to several classification systems based on histology, inheritance patterns or clinical phenotypes.^3,6^ For primary care doctors, clinical phenotyping is more relevant in daily practice. It divides lesions by their patterns and distributions: diffuse, focal, striate and punctate.^3,7^ Beyond each categories lies a wide range of HPPK subtype.

Identifying associated syndromes is more important than subclassification, particularly for frontliners. HPPK may involve other organ, and some association can be life-threatening.^1,7^ Although our patient showed no syndromic features like deafness, cardiomyopathy, or dental abnormalities, recognising these associations is essential. Cardiovascular and dental screening is compulsory, as complications include dilated cardiomyopathy and periodontitis.^3^ HPPK is also linked to oesophageal cancer and squamous cell carcinoma.^1,8^ HPPK may associated with hearing loss, it can reduce the quality of life. Other extracutaneous features, including ichthyosis; woolly hair; ectodermal dysplasia; peeling skin with leukonychia, acral punctate keratoses, cheilitis and knuckle pads; and guttate hypopigmentation. This involvement is classified as syndromic HPPK.^1,7^

**Figure 5:**
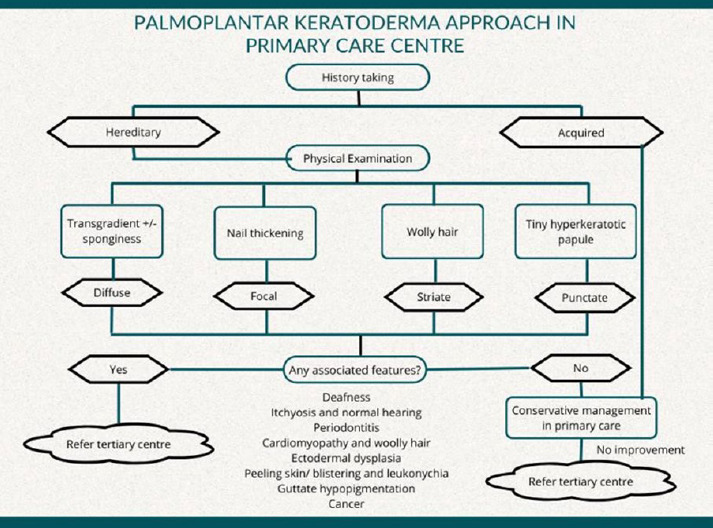
Proposed algorithm ofthe PPK approacli in primary care settings.

In the primary care setting, case evaluation varies by practitioner’s background. Herein, we propose local guidelines (Figure 5) to facilitate early detection and timely management in resource-limited settings.

Diagnosing PPK requires a comprehensive assessment to identify causes and associated syndromes. In our case, early onset, transgrediens distribution and similar findings in her father and brother supported the diagnosis. Important points included the age of onset, family history, seasonal variations and hair or nail abnormalities. Other relevant factors are heart failure symptoms, deafness, dental loss, photosensitivity and consanguinity marriages must also be documented.^5^ Extracutaneous signs should prompt referral for skin biopsy and genetic testing. A biopsy can distinguish acquired from HPPK.^1,9^ Genetic testing identifies causative mutations^10^ and hereditary pattern.^3^ Certain types of PPK exhibit autosomal dominant, while others follow a recessive pattern with syndromic features.^3^ Although unavailable in primary care settings, genetic testing should be a referral point to tertiary care settings, particularly for genetic counselling. Nevertheless, the treatment can initiate without delay.

Treating HPPK is challenging, as the condition is lifelong^7^ and severely impacts the quality of life.^11^ Complications include infections, pain, impaired mobility, fissures and ulcers, and joint stiffness if left untreated.^1^ HPPK also leads to social and psychological distress.^11^ No therapy offers lasting effects, recurrence is frequent after discontinuation of treatment.^1^ Patients must be educated on continuous hand and foot care.

Treatment rims to control hyperkeoatosis, improve function and cosmetic appearance and reduce pain. Table 2 shows the treatment options offered.^1,3,7,12^ Failed first line topical treatment warrants a tertiary referral. Regular follow-up is essential. Secondary infections require appropriate topical or systemic antimicrobial.^1,3^

**Table 2:** Treatment options offered to the patient.

Care level	Treatment option	Notes
**Primary care**	**Topical keratolytic**^1,3,7^ - Salicylic acid 5%-10%	- First-line treatment to soften and exfoliate the thickened skin - Daily or weekly soaked baths, followed by mechanical scales _removal_^1,3,7^
	**Emollients**^13^ - Aqueous cream	- Maintain skin hydration and integrity
**Tertiary care**	**Topical keratolytic**^1^ - Urea 10% cream	- First-line treatment to soften and exfoliate the thickened skin
	**Topical retioniods**^1,3,7^ - Tretinoin cream 0.05%	- Helps in reducing keratinisation - Use with caution due to potential skin irritation
	Emollient^7^ - Petroleum-based product (e.g. Vaseline)	- Maintain skin hydration and integrity
	Systemic retinoid^1,3,7^ - Acitretin	- Used for severe or refractory cases - Requires close monitoring due to side effects, including teratogenicity,^12^ and contraindication in pregnancy

Beyond dermatologie care, syndromic PPK requires an individualised and multidisciplinary approach based on the severity of their associated features. Early detection allows parental counselling, referral to tertiary care centres and timely intervention.^1^ Parents must anticipate the chronicity of keratoderma and its long-term impacts on their children’s well-being. Primary care physicians must provide ongoing counselling to support parents and patients. Promoting awareness helps reduce parental distress and support better coping mechanisms.

In this case, the patient’s symptoms began at the age of 2 years, coinciding with walking. This is concurrent with hyperkeratosis involving both palm and soles. A positive family history and the absence of extracutaneous features or triggering factors suggested for transgrediens diffuse HPPK. This reinforces the value of structured assessment in primary care settings. Subsequently, with appropriate keratolytic treatment, the patient’s condition significantly improved.

A similar Hong Kong case series described three HPPK patient with diffuse hyperkeratosis since infancy.^14^ Two had Meleda disease, characterised by well-demarcated transgrediens lesions, nail dystrophy, and knuckle pads, while one had Nagashima-type PPK, a milder form with spongy changes after water exposure. In contrast, our patient exhibited diffuse transgrediens hyperkeratosis without mutilating features or nail involvement, and with a strong family history-suggestive of non-syndromic diffuse HPPK. Another case report described punctate HPPK in a 36-year-old woman with autosomal dominant inheritance and late-onset papular lesions on the palms and soles.^13^ These comparisons reinforce the clinical value of phenotype-based differentiation. Despite resource constraints, careful assessment of onset, lesion morphology, distribution and family history - as demonstrated in our case - can lead to a presumptive diagnosis and timely intervention at the primary care level.

## Conclusion

This case underscores the importance of distinguishing hereditary from acquired PPK. In primary care settings where genetic testing is unavailable, diagnosis can rely on clinical evaluation, dermatological assessment, and family history. Without early treatment, HPPK can lead to disastrous physical, social and psychological complications. Preparing parents for its chronic nature and ensuring continuous follow-up are essential for an optimal long-term outcome.
